# Systemic Idiopathic Polyarteritis Nodosa Mimicking Cryoglobulinemic Vasculitis: A Diagnostic Challenge

**DOI:** 10.7759/cureus.112277

**Published:** 2026-07-08

**Authors:** Abdus Saboor, Helmi A Ahmed, Fathi Abourawi

**Affiliations:** 1 Diabetes and Endocrinology, Diana, Princess of Wales Hospital, Grimsby, GBR

**Keywords:** anca-negative vasculitis, cryoglobulinemia vasculitis, leukocytoclastic vasculitis (lcv), medium vessel vasculitis, medium-vessel vasculitis, peripheral sensory neuropathy, polyarteritis nodosa (pan), testicular pain, vasculitic neuropathy

## Abstract

Polyarteritis nodosa (PAN) is an uncommon systemic vasculitis involving predominantly medium-sized arteries. Its heterogeneous presentation and relapsing-remitting course often lead to diagnostic delay. We report a 68-year-old male with a 15-month history of progressive peripheral neuropathy, recurrent vasculitic rash, renal impairment, and testicular pain. Laboratory investigations revealed elevated inflammatory markers, positive rheumatoid factor, type II cryoglobulins, and negative antineutrophil cytoplasmic antibody (ANCA). Complement levels were normal on repeated testing, and urinalysis showed isolated proteinuria without red blood cells or casts, arguing against other differentials like cryoglobulinemic vasculitis and glomerulonephritis. Skin biopsy demonstrated leukocytoclastic vasculitis. After excluding secondary causes, including hepatitis B, ANCA-associated vasculitis (granulomatosis with polyangiitis and microscopic polyangiitis), IgA vasculitis, and cryoglobulinemic vasculitis, a diagnosis of systemic idiopathic PAN was established. Cyclophosphamide was considered but avoided due to pre-existing renal impairment. The patient was treated with high-dose prednisolone for induction, followed by low-dose azathioprine as a steroid-sparing agent, with subsequent clinical improvement. This case illustrates the diagnostic difficulty of distinguishing PAN from cryoglobulinemic vasculitis when overlapping serological features are present, and the consequences of delayed recognition on organ function.

## Introduction

Polyarteritis nodosa (PAN) is a systemic necrotizing vasculitis that primarily affects medium-sized arteries, sparing small vessels and glomeruli [1]. According to the 2012 Revised International Chapel Hill Consensus Conference nomenclature, PAN is classified among medium-vessel vasculitides and is characteristically antineutrophil cytoplasmic antibody (ANCA)-negative [2]. The disease is rare, with an estimated annual incidence of two to nine cases per million [3]. Clinical manifestations are heterogeneous, resulting from vascular inflammation and tissue ischemia. Common features include constitutional symptoms, cutaneous involvement, peripheral neuropathy, musculoskeletal complaints, and renal impairment [4]. The relapsing-remitting nature and non-specific early symptoms frequently result in delayed diagnosis. Importantly, the differential diagnosis of PAN includes several conditions with overlapping features, notably ANCA-associated vasculitides (granulomatosis with polyangiitis and microscopic polyangiitis), cryoglobulinemic vasculitis, and IgA vasculitis, all of which may present with purpura, neuropathy, and renal involvement [3]. We describe a patient with systemic PAN whose fluctuating multisystem symptoms and serological findings mimicking cryoglobulinemic vasculitis contributed to a prolonged diagnostic course.

## Case presentation

A 68-year-old male presented with a 15-month history of progressive constitutional and neurological symptoms. His primary complaints included persistent fatigue, numbness of both feet, intermittent calf pain on walking, a recurrent rash beginning on the soles and spreading to the lower limbs and abdomen, and recurrent abdominal pain radiating to the perineum and testes. The rash was described by the patient as pruritic, which is atypical for PAN-associated cutaneous vasculitis and initially broadened the differential diagnosis to include other vasculitic processes such as cryoglobulinemic vasculitis [3,5]. The symptoms were intermittent, with episodes of rash and pain resolving over several weeks before recurring. He denied weight loss, sinus symptoms, respiratory complaints, or fevers. His past medical history included chronic obstructive pulmonary disease and an incidental hepatic hemangioma. He had no history of hepatitis B or C, HIV, alcohol use, or smoking.

**Figure 1:**
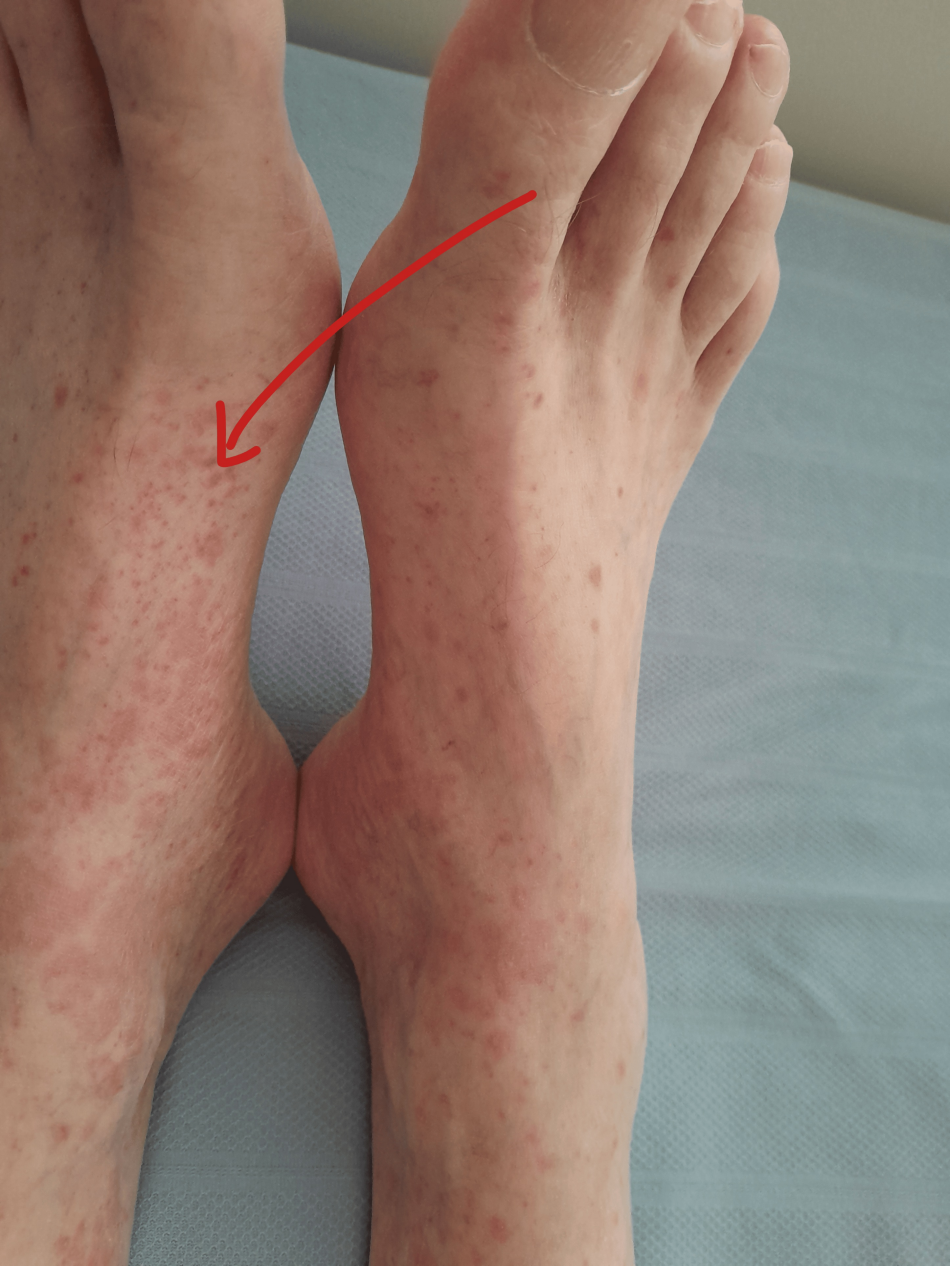
Non-blanching purpuric vasculitic rash involving the feet.

**Figure 2:**
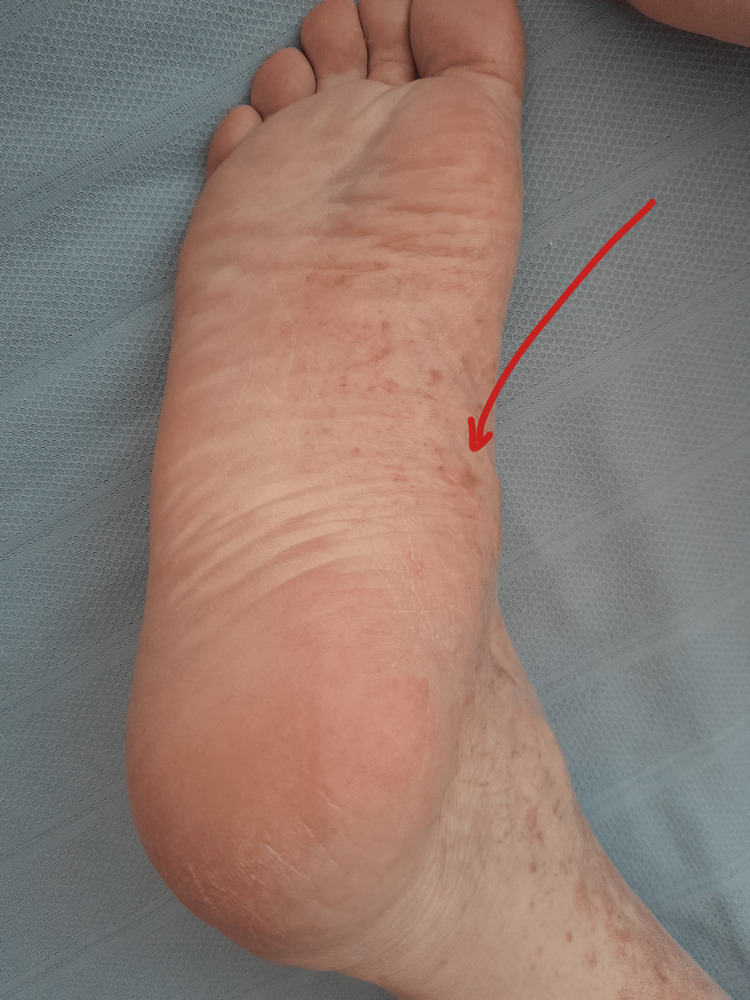
Non-blanching purpuric vasculitic rash involving the sole of the foot.

**Figure 3:**
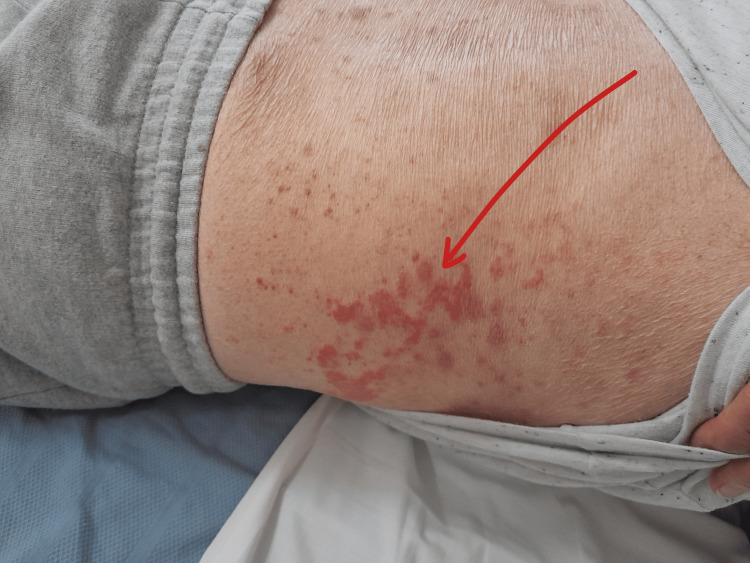
Non-blanching purpuric vasculitic rash involving the abdomen.

On examination, the patient was hemodynamically stable with a blood pressure of 127/85 mmHg. Cardiovascular, respiratory, and abdominal examinations were unremarkable. Neurological examination revealed reduced touch and temperature sensation in both lower limbs in a length-dependent pattern consistent with peripheral neuropathy; proprioception and vibration sense were preserved, and motor power was intact (5/5 in all limbs). Dermatological examination demonstrated a non-blanching purpuric vasculitic rash involving the soles of the feet, extending to the lower limbs, abdomen, and back (Figures 1-3).

Laboratory investigations revealed renal impairment (estimated glomerular filtration rate (eGFR) = 30-33 mL/min, creatinine = 178 µmol/L, urine protein-creatinine ratio = 67 mg/mmol) and elevated inflammatory markers (C-reactive protein (CRP) = 33 mg/L, erythrocyte sedimentation rate (ESR) = 58 mm/hr). Urinalysis showed proteinuria only, with no red blood cells or casts, making active glomerulonephritis unlikely. Liver function tests showed a cholestatic pattern (alkaline phosphatase (ALP) = 419 U/L, gamma-glutamyl transferase (GGT) = 430 U/L) with hypoalbuminemia (29 g/L). Immunological studies demonstrated markedly elevated rheumatoid factor (4521 IU/mL), positive type II cryoglobulins, positive antinuclear antibody (ANA) (homogeneous and speckled pattern), and negative ANCA (myeloperoxidase/proteinase 3). Complement levels were measured on two occasions: initial C3 was 1.17 g/L, and C4 was 0.30 g/L; repeat testing showed C3 of 1.31 g/L and C4 of 0.22 g/L (reference ranges: C3 = 0.9-1.8 g/L; C4 = 0.1-0.4 g/L). Both sets of results were within normal limits, which was a significant finding in the differential diagnosis (see Discussion). Viral serology for hepatitis A, B, C, and HIV was negative. Nerve conduction studies showed predominantly sensory peripheral neuropathy. Skin punch biopsy demonstrated leukocytoclastic vasculitis. CT aortogram showed no aneurysms or arterial abnormalities. PET scan revealed no evidence of malignancy or large-vessel vasculitis (Table 1).

**Table 1:** Summary of key laboratory investigations. eGFR: estimated glomerular filtration rate; MPO-ANCA: myeloperoxidase-antineutrophil cytoplasmic antibody; PR3-ANCA: proteinase 3-antineutrophil cytoplasmic antibody.

Investigation	Result	Reference range
Creatinine (initial/repeat)	178 µmol/L/145 µmol/L	64-104 µmol/L
eGFR (initial/repeat)	33 mL/min/42 mL/min	>90 mL/min
Urine protein-creatinine ratio	67 mg/mmol	0-30 mg/mmol
Urine RBC/casts	Absent	Absent
C-reactive protein (CRP) (initial/repeat)	33 mg/L/5.3 mg/L	0-5 mg/L
Erythrocyte sedimentation rate (ESR) (initial/repeat)	58 mm/hr/29 mm/hr	<20 mm/hr
Alkaline phosphatase (ALP)	419 U/L	30-130 U/L
Gamma-glutamyl transferase (GGT)	430 U/L	10-71 U/L
Albumin	29 g/L	35-50 g/L
Complement C3 (initial/repeat)	1.17/1.31 g/L	0.9-1.8 g/L
Complement C4 (initial/repeat)	0.30/0.22 g/L	0.1-0.4 g/L
Rheumatoid factor	4521 IU/mL	0-14 IU/mL
Antinuclear antibody (ANA)	Positive (homogeneous + speckled)	Negative
Cryoglobulins (Type II)	Positive	Negative
MPO-ANCA	<3.2 U	0-20 U
PR3-ANCA	<2.3 U	0-20 U
Hepatitis A, B, C	Negative	Negative
HIV	Negative	Negative

Diagnosis

Based on the clinical presentation of peripheral neuropathy, vasculitic rash, renal impairment, testicular pain, elevated inflammatory markers, negative ANCA, and exclusion of secondary causes, including hepatitis B infection, a diagnosis of systemic idiopathic PAN was made [1,2]. The patient fulfilled five of the 10 1990 American College of Rheumatology classification criteria for PAN: testicular pain, myalgia and leg pain, peripheral neuropathy, renal impairment, and histological evidence of vasculitis [6]. The presence of markedly elevated rheumatoid factor and positive type II cryoglobulins warranted careful exclusion of cryoglobulinemic vasculitis; however, the negative hepatitis C serology, repeatedly normal complement C3 and C4 levels, absence of red blood cells or casts on urinalysis (indicating low suspicion for glomerulonephritis in the absence of renal biopsy), presence of testicular pain, and the overall clinical picture favored PAN over cryoglobulinemic vasculitis [7]. Other differential diagnoses considered included ANCA-associated vasculitides (excluded by negative ANCA and absence of pulmonary or upper respiratory tract involvement) and IgA vasculitis (less likely given the patient's age, absence of IgA deposits, and presence of testicular pain more characteristic of PAN) [3].

Management and outcome

The patient was commenced on high-dose oral prednisolone (1 mg/kg/day) in line with current treatment guidelines for severe PAN [8]. Cyclophosphamide was considered as first-line induction therapy given the presence of organ-threatening disease (renal impairment, peripheral neuropathy); however, cyclophosphamide requires dose reduction in renal impairment due to decreased excretion and increased risk of toxicity [8,9]. In this patient, the combination of an eGFR of 30-33 mL/min, advanced age, and the clinical team's assessment of the risk-benefit profile led to the decision to initiate azathioprine at a low dose as a steroid-sparing agent rather than cyclophosphamide for induction [8,10]. It is acknowledged that cyclophosphamide remains the standard induction agent for severe PAN and that the evidence supporting azathioprine as primary induction therapy is less robust; azathioprine has primarily demonstrated efficacy as a maintenance agent and in vasculitis without poor prognosis factors [10]. Following initiation of glucocorticoids, the patient demonstrated clinical improvement, particularly in renal function and neuropathic symptoms. Although some renal impairment was irreversible, further disease progression was halted. The patient remains on close follow-up given the high relapse rate reported in PAN [4].

## Discussion

PAN is an uncommon but potentially serious vasculitis with diverse clinical manifestations [1]. In this patient, several recognized features of PAN were present, including subacute onset, multisystem involvement, and relapsing-remitting course, which collectively contributed to a 15-month diagnostic delay.

Peripheral nervous system involvement is one of the most frequent manifestations of PAN, affecting 50-75% of patients [11]. The classic presentation is mononeuritis multiplex due to inflammation and ischemia of the vasa nervorum, though confluent mononeuropathies may mimic symmetric polyneuropathy as observed in this case [11]. In this patient, the predominantly sensory neuropathy with loss of touch and temperature sensation but preserved proprioception and motor function is consistent with small-fiber involvement from vasa nervorum ischemia. Skin manifestations are common early clues and include livedo reticularis, subcutaneous nodules, ulcerations, and purpura [3]. The patient's description of pruritus associated with the rash is noteworthy, as PAN-associated cutaneous lesions are not typically pruritic; purpura, livedo, and nodules are the most frequent skin findings in systemic PAN [5]. This atypical feature initially broadened the differential to include cryoglobulinemic vasculitis, in which purpuric lesions may be triggered by cold exposure and can be associated with pruritus [7]. Testicular pain, resulting from orchitis due to testicular artery vasculitis, is a characteristic but often overlooked symptom relatively specific to PAN among systemic vasculitides [3]. Renal involvement in PAN typically manifests as ischemic nephropathy with renovascular hypertension and renal insufficiency, rather than glomerulonephritis, which distinguishes it from ANCA-associated vasculitides [1,2].

A central diagnostic challenge in this case was distinguishing PAN from cryoglobulinemic vasculitis, given the presence of positive type II cryoglobulins and markedly elevated rheumatoid factor. Cryoglobulinemic vasculitis classically presents with the triad of purpura, arthralgias, and nephritis, and is most commonly associated with hepatitis C virus infection [7]. Key laboratory features that distinguish cryoglobulinemic vasculitis include low complement C4 levels (frequently consumed via classical pathway activation) and the presence of membranoproliferative glomerulonephritis with an active urinary sediment [7]. In this patient, complement C3 and C4 levels were normal on two separate occasions, which argues strongly against active cryoglobulinemic vasculitis, as low C4 is present in 70-90% of cases of mixed cryoglobulinemia syndrome [7]. Furthermore, urinalysis demonstrated isolated proteinuria without red blood cells or casts, indicating low suspicion for glomerulonephritis; however, it is acknowledged that renal biopsy was not performed, and therefore glomerulonephritis cannot be definitively excluded. The negative hepatitis C serology, presence of testicular pain (a feature characteristic of PAN but not of cryoglobulinemic vasculitis), and the overall clinical pattern collectively supported PAN as the primary diagnosis [3,7]. Cryoglobulins can be detected in various inflammatory and autoimmune conditions, and their presence alone does not establish a diagnosis of cryoglobulinemic vasculitis [7].

An important finding in this case was the presence of leukocytoclastic vasculitis on skin punch biopsy. By the strict Chapel Hill Consensus Conference 2012 definitions, PAN should not involve small vessels [2]. However, in clinical practice, superficial skin biopsies may not sample the deeper dermal vessels where medium-vessel vasculitis is typically found. The 2021 American College of Rheumatology (ACR)/Vasculitis Foundation guideline conditionally recommends obtaining a deep-skin biopsy specimen reaching medium-sized vessels of the dermis over a superficial punch biopsy to aid in establishing a diagnosis of PAN [8]. This represents a limitation of the histological evidence in this case, and a deep biopsy should be attempted when PAN is suspected.

The CT aortogram in this case showed no aneurysms or medium-vessel abnormalities. While conventional catheter-based angiography remains the gold standard for detecting microaneurysms in PAN due to its superior resolution, CT angiography is increasingly used as a less invasive alternative [8]. However, CT angiography has limited sensitivity for detecting small microaneurysms in distal arterial branches, and a negative CT angiogram does not exclude PAN [8]. The 2021 ACR/Vasculitis Foundation guideline recommends that, in patients with a negative CT or magnetic resonance angiogram result but high clinical suspicion for abdominal involvement, conventional angiography should be considered [8]. In this case, the absence of angiographic abnormalities does not refute the diagnosis, as aneurysms are not universally present in PAN, and the diagnosis was supported by multiple clinical, laboratory, and histological criteria.

Diagnosing PAN can be difficult in practice due to the absence of specific serological markers and fluctuating symptoms [1,12]. While biopsy-proven necrotizing arteritis of medium-sized vessels is the gold standard, diagnosis often relies on a combination of clinical, laboratory, imaging, and histological findings [12]. The ACR 1990 classification criteria were designed for research purposes and have recognized limitations when used diagnostically, including poor specificity in distinguishing PAN from ANCA-associated vasculitides [6]. Provisional seven-item diagnostic criteria have been proposed with improved sensitivity (92.3%) and specificity (91.7%), though these require further validation [12].

Management decisions in PAN are largely determined by disease severity. The 2021 ACR/Vasculitis Foundation guideline recommends cyclophosphamide and glucocorticoids for severe PAN, defined as vasculitis with life- or organ-threatening manifestations, including renal disease, mononeuritis multiplex, or mesenteric ischemia [8]. Cyclophosphamide dose should be reduced for renal impairment and age, as these patients are at increased risk for infection and toxicity [9]. For patients unable to tolerate cyclophosphamide, other non-glucocorticoid immunosuppressive agents such as azathioprine or methotrexate combined with glucocorticoids are recommended over glucocorticoid monotherapy [8]. In this case, azathioprine was selected as a steroid-sparing agent given the patient's reduced renal function and advanced age. It is important to acknowledge that the evidence for azathioprine as primary induction therapy in severe PAN is less robust than for cyclophosphamide; azathioprine has primarily demonstrated efficacy as a maintenance agent in systemic necrotizing vasculitides without poor prognosis factors and provides a glucocorticoid-sparing effect [10]. The decision to use azathioprine over cyclophosphamide in this case was a clinical judgment balancing the risks of cyclophosphamide toxicity against the potential for suboptimal disease control.

## Conclusions

This case underlines the need to consider PAN in patients presenting with peripheral neuropathy, vasculitic rash, renal dysfunction, and systemic symptoms, particularly when ANCA is negative. The presence of positive cryoglobulins and elevated rheumatoid factor should prompt careful exclusion of cryoglobulinemic vasculitis; normal complement levels (especially C4) and absence of an active urinary sediment are important distinguishing features. Moreover, when suspecting PAN, a deep skin biopsy should be attempted instead of a superficial punch biopsy, and a negative CT angiogram should be interpreted with caution, given its limited sensitivity for small microaneurysms. Early recognition and prompt treatment with glucocorticoids, combined with appropriate immunosuppressive therapy tailored to disease severity and patient factors, can prevent irreversible organ damage and improve outcomes.
